# Fear of disease in patients with epilepsy – a network analysis

**DOI:** 10.3389/fneur.2024.1285744

**Published:** 2024-03-07

**Authors:** Xiaoxiao Yin, Shan Niu, Qun Yu, Yejing Xuan, Xiuqin Feng

**Affiliations:** Nursing Department, The Second Affiliated Hospital Zhejiang University School of Medicine (SAHZU), Hangzhou, China

**Keywords:** epilepsy, fear, psychosocial, network analysis, Chinese patients

## Abstract

**Background:**

Disease-related fear among patients with epilepsy has significantly impacted their quality of life. The Disease-Related Fear Scale (D-RFS), comprising three dimensions, serves as a relatively well-established tool for assessing fear in these patients. However, certain problems potentially exist within the D-RFS’s attribution of items, and its internal structure is still unclear. To establish an appropriate dimensional structure and gain deeper comprehension of its internal structure—particularly its core variables—is vital for developing more effective interventions aimed at alleviating disease-related fear among patients with epilepsy.

**Methods:**

This study employed a cross-sectional survey involving 609 patients with epilepsy. All participants underwent assessment using the Chinese version of the D-RFS. We used exploratory network analysis to discover a new structure and network analysis to investigate the interrelationships among fear symptom domains. In addition to the regularized partial correlation network, we also estimated the node and bridge centrality index to identify the importance of each item within the network. Finally, it was applied to analyze the differences in network analysis outcomes among epilepsy patients with different seizure frequencies.

**Results:**

The research findings indicate that nodes within the network of disease-related fear symptoms are interconnected, and there are no isolated nodes. Nodes within groups 3 and 4 present the strongest centrality. Additionally, a tight interconnection exists among fear symptoms within each group. Moreover, the frequency of epileptic episodes does not significantly impact the network structure.

**Conclusion:**

In this study, a new 5-dimension structure was constructed for D-RFS, and the fear of disease in patients with epilepsy has been conceptualized through a network perspective. The goal is to identify potential targets for relevant interventions and gain insights for future research.

## 1 Introduction

Due to the recurrent and unpredictable nature of epilepsy, patients with epilepsy experience psychological symptoms more frequently than the general population and individuals with other chronic diseases (1, 2). For instance, anxiety disorders are highly prevalent as psychiatric comorbidities in epilepsy (3), with an average prevalence of 32.6% for clinically significant anxiety symptoms among individuals newly diagnosed with epilepsy (4). Notably, fear constitutes a notable symptom frequently linked to generalized anxiety disorder (5, 6). Disease-related fears are connected to the unfavorable ramifications of the condition, encompassing seizures, the persistent nature of the ailment, the therapeutic regimen, as well as the psychological aftermath of the sickness (7, 8). This includes sensations of distinctiveness, deviance from the norm, discomfort, self-endangerment, and posing risks to both oneself and others (9). This negative emotion can potentially trigger “overcompensation behaviors” in patients with epilepsy, as they attempt to gain control over or alleviate their concerns related to epilepsy. These behaviors may include avoiding social activities, restricting daily activities to reduce the risk of injury, seeking frequent medical attention to ensure their health status, overly relying on family or friends, avoiding solitude, and so on (4, 6, 7). While these behaviors might offer some short-term emotional relief, in the long run, they could limit the patients’ quality of life, social interactions, and daily activities. Therefore, understanding the underlying mechanisms of these emotions is crucial for effectively helping patients with epilepsy manage their condition and enhance their quality of life.

There are ongoing uncertainties regarding the underlying mechanisms of disease-related fear in patients with epilepsy (10). Considering the specific needs of different individuals, identifying these mechanisms is crucial for devising targeted intervention measures aimed at reducing the perception of disease-related fear among epilepsy patients, assisting them in avoiding long-term psychological consequences, and enhancing their quality of life. While research on the fear of cancer recurrence among cancer patients exists (11, 12), its applicability is limited in the context of epilepsy due to the unique characteristics of the condition. There is a current gap in the literature regarding the components and interrelationships of disease-related fear in epilepsy patients.

Network analysis is a promising statistical methodology used to address the issues. Built upon data, it circumvents *a priori* assumptions about causal relationships between variables and instead conducts mathematical analysis and visual representation of variable relationships (13, 14). Network analysis introduces a fresh approach to conceptualizing psychological structures, portraying them as complex systemic phenomena arising from interactions among their components. Within psychological networks, nodes represent psychological variables such as emotions, symptoms, attitudes, while edges represent relationships between variables, and groups represent groups of related psychological variables (15). In this context, components cease to be mere passive indicators reflecting concepts; rather, they become active indicators playing a role in the process of concept emergence. Hence, given the intricacy of fear symptoms, it’s reasonable to regard them as complex systemic phenomena arising from interactions across various dimensions. Furthermore, network analysis can provide metrics for node and bridge centrality, evaluating the significance and control of each node. When nodes with high centrality are activated, they are likely to propagate activation across the network by connecting to other nodes through edges (16). This offers essential potential targets for relevant intervention measures (17). In recent years, network analysis has garnered widespread attention and application across various domains of psychology, such as depression (18), anxiety (19), acute stress reactions (20), eating disorders symptoms (21), and more. Additionally, some items in the Chinese version of the D-RFS may not be suitable for their factor assignments (22), suggesting the possibility of a better structural model.

Therefore, this study adopted exploratory network analysis to determine a new structure, and applied network analysis to investigate the fine-grained relationships among fear symptoms in patients with epilepsy. We developed a network model to explore the interconnected pathways of fear symptoms and assessed node and bridge centrality, laying a theoretical foundation for identifying effective symptom targets for psychological interventions.

## 2 Methods

### 2.1 Study design and settings

This study was a cross-sectional study conducted at the specialist epilepsy clinic and the epilepsy center ward in a general tertiary hospital in Hangzhou, Zhejiang province, China from January to June 2023. The selection criteria included the following: (1) adults (18 years or above), (2) a diagnosis of epilepsy made in the past 6 months based on a review of medical records and confirmed by treating physicians, (3) the ability to read and articulate in Chinese, and (4) not undergoing treatment for psychiatric disorders. Ethical approval for this study was obtained from the hospital, and electronic informed consent was obtained online from all participants. A total of 700 questionnaires were distributed, with 609 valid responses, including 50 paper and 650 online versions. Seven paper surveys were excluded due to over three blank items. No omissions were found in the online surveys as all questions were mandatory. However, 27 questionnaires were deemed invalid due to completion in less than two minutes. In addition, 57 respondents failed to select the correct options on two specific questions, rendering their questionnaires invalid.

### 2.2 Measurements

The validated Chinese version of the Disease-Related Fear Scale (D-RFS) was used to assess epilepsy-related fear (22). Our research team translated and validated the scale, originally developed by Shamsalinia et al. (7). It has shown reliability and validity where it has been adapted and subjected to the appropriate validation (23, 24). The D-RFS consists of 27 items divided into 3 factors: fear of seizure consequences, fear of poor epilepsy management, and fear of social restrictions. All items are forward-scored using a 4-point Likert response format from very low (1) to very high (4). The total score of the D-RFS ranges from 0 to 100, with higher scores indicating greater severity of disease-related fear. Fear of seizure consequences refers to the fear of experiencing accidental harm during a seizure (items 1, 2, 3, 4, 6, 8, and 9). Fear of poor epilepsy management refers to the fear of physical or emotional harm resulting from poor management of epilepsy (items 5, 7, 10, 11, 12, 13, 14, 17, 18, 19, 20, 21, 22, 23, and 24). Fear of social restrictions refers to the fear of a lack of or limited personal closeness with friends and family, as well as reduced social connections with others due to epilepsy (items 15, 16, 25, 26, and 27). The D-RFS had a Cronbach’s alpha of *α* = 0.96 in this sample (22). Additionally, participant sociodemographic data (such as age, gender, marital status, education level, and income) and medical information (such as epilepsy type, time of diagnosis, treatment status, and frequency of seizures) were collected through self-report.

### 2.3 Statistical analyses

For the purposes of conducting descriptive statistics, exploratory network analysis, confirmatory factor analysis, network estimation, network accuracy and stability, and bridges centrality, this study utilized the psych, igraph, lavaan, bootnet, and networktools packages within the R Studio 4.2.0.

#### 2.3.1 Descriptive statistics and one-way analysis of variance

Descriptive analyses were performed using the describeBy function of the psych packages, reporting the mean and standard deviation (SD) for continuous variables, and the percentage and frequency for categorical variables. In statistical inference, the significance level *α* was set at 0.05, and a two-tailed *p*-value was used for hypothesis testing. This study used the Companion to Applied Regression package in R studio to perform one-way Analysis of Variance (ANOVA) to analyze the impact and statistical significance of sociodemographic features such as age, gender, and education level, as well as clinical characteristics including type of illness, duration of disease, and severity of symptoms, on the disease-related fear among patients with epilepsy.

#### 2.3.2 Reliability and structure validity

##### 2.3.2.1 Exploratory network analysis

Initially, the Kaiser–Meyer–Olkin (KMO) test and Bartlett’s test of sphericity were used to determine the sampling adequacy for factor analysis. KMO values above 0.8 and significant results from Bartlett’s test both confirm the suitability of the data for factor analysis (25).

In R Studio, the estimateNetwork function from the EGAnet package was utilized in conjunction with the EBICglasso method to build a network model. This combined approach employed the Extended Bayesian Information Criterion to estimate the associative structure within high-dimensional data, establishing the connections between variables. Once the network model estimation was complete, the code adjusted all negative-weight edges by converting them to their absolute values to meet the requirements of group detection algorithms. Further, using the igraph package, the cluster_walktrap method was applied to perform group detection on the constructed graphical network. The Walktrap method, a graph theory-based group discovery algorithm, simulated random walks within the network to identify boundaries between nodes, pinpointing clusters of tightly connected nodes that formed groups within the network. Finally, the network model was visualized using the qgraph function. In the resultant network graph, nodes were colored and grouped according to their group membership, providing a clear visual representation of the different groups and their relationships. This visualization tool greatly facilitated the identification and analysis of the network’s group structure. In the composition of D-RFS, network nodes represented a multitude of independent items, which were systematically woven into unique group networks based on the relational nature of the projects they carried. Meanwhile, the edges forming bridges between nodes mapped the interactions among these entries.

##### 2.3.2.2 Confirmatory factor analysis

The lavaan package was utilized for CFA to ascertain the factor structure of the D-RFS and assess the model’s fit. In the evaluation of the CFA model’s fit, several indices were considered, including the chi-square Index (*χ*^2^ and *p*-value), the standardized root mean square residual (SRMR), the root mean square error of approximation (RMSEA), and fit indices such as the Incremental Fit Index (IFI), Comparative Fit Index (CFI), Tucker-Lewis Index (TLI), Relative Fit Index (RFI), and the Normed Fit Index (NFI). An acceptable model fit was indicated by IFI, CFI, TLI, RFI, and NFI values of 0.80 or above, and SRMR and RMSEA values of 0.08 or below (26). An excellent model fit was signified by IFI, CFI, TLI, RFI, and NFI values of 0.90 or above, and SRMR and RMSEA values of 0.05 or below (26). Additionally, CFA was conducted to examine the model structure identified through network exploratory analysis and to compare this to CFA outcomes derived following traditional exploratory analysis. This comparative analysis played a key role in establishing the structure’s stability and the validity of the theoretical assumptions, thereby bolstering the model’s robustness and trustworthiness. Through this method, a greater comprehension of the items’ interrelationships was achieved, ensuring the research hypotheses were substantiated, and providing a firm basis for the study’s conclusions.

##### 2.3.2.3 Convergent validity

Convergent validity was evaluated through the Average Variance Extracted (AVE), a metric representing the proportion of variance that a construct captures as opposed to variance caused by measurement error (values exceeding 0.5 are deemed acceptable, while those surpassing 0.7 are considered exemplary) (27).

##### 2.3.2.4 Reliability

Reliability was evaluated by calculating the Cronbach’s alpha for each item upon its removal, as well as the aggregate Cronbach’s alpha coefficients. Alpha values above 0.7 were considered acceptable (28). Additionally, we measured the reliability using the Macdonald’s Omega (*ω*) coefficient. The Macdonald’s Omega coefficient offers numerous benefits over the widely used Cronbach’s alpha, such as improved precision and less restrictive, more pragmatic assumptions (29).

#### 2.3.3 Network estimation

##### 2.3.3.1 Network structure

The group results derived from exploratory network analysis were conducted within R program using the bootnet (30) and qgraph (31) packages. The Graphical Gaussian Model (GGM) with the graphic least absolute shrinkage and selection operator (LASSO) and the Extended Bayesian Information Criterion (EBIC) model were used to shrink minor edges to zero weight (32). To ensure accurate correlation estimation, the corMethod parameter was set to “cor_auto,” allowing the function to automatically select the appropriate correlation method based on the data type (33). The network nodes symbolize the distinct components within the D-RFS. Based on the subordination of items represented by the nodes, the nodes were organized into distinct item groups. The edges connecting the nodes represent the partial correlation coefficients between the items (34). An existing edge between two nodes signifies a present relation between the corresponding items, considering all other nodes in the network. Thicker edges indicate stronger underlying association between the items. Highly correlated nodes appear closely together in the figure, and nodes mapped closer to the center have more correlations with other items than those mapped farther from the center. The color of the edge indicates the direction of the correlations (e.g., blue edges represented positive correlations; red edges represented negative correlations).

##### 2.3.3.2 Central symptoms

To highlight which symptoms may have the greatest overall impact on the network, the strength, betweenness, and closeness centrality indices were calculated. However, recent studies have shown that strength is the most reliable centrality index, whereas betweenness and closeness centrality indices are not suitable for assessing node importance in psychological networks (35, 36). Therefore, the strength centrality of each node was computed to evaluate and quantify their relative importance across the entire network. Furthermore, the expected influence (EI) of nodes in the network was evaluated by considering the sum of edge weights between a node and its immediate neighbor nodes, including both positive and negative associations. In this study, centrality indices were computed using the “centralityPlot” function.

##### 2.3.3.3 Bridges centrality

Furthermore, by measuring bridge strength and bridge expected influence (BEI), the mediation and influence of nodes in connecting different groups were evaluated (37). The bridge centrality index represents bridging nodes that have the strongest connections to another group. The distinction between node centrality index and bridge centrality index lies in the latter assessing the associations between two nodes, each belonging to different groups. In short, bridge centrality reflects the importance of a node in connecting two distinct groups. And it is advisable to keep only the most central 20% of bridges in the network (37). The bridge centrality was estimated by R package networktools (38).

#### 2.3.4 Network accuracy, and stability

We employed a comprehensive set of methods to examine the stability and accuracy of the network, including case-dropping bootstrap tests for centrality stability and bootstrap confidence intervals (CIs) to assess the accuracy of network edges. Firstly, we employed a non-parametric bootstrap to estimate the 95% CIs of edge values, evaluating the accuracy of edge weights. Wider CIs indicate reduced precision in estimating edge weights, while narrower CIs suggest a more reliable network. Secondly, we utilized a case-dropping subset bootstrap to calculate the correlation stability coefficient (CS-C) through 500 permutations. CS-C measures the robustness of the correlation between original indices and those based on a case-subset network. Generally, CS-C should not be lower than 0.25 and preferably above 0.5 (32). Additionally, within this network analysis, we conducted bootstrap difference tests to examine differences in network properties. This test relies on 95% CIs to determine if significant differences exist in two-node centrality indices or edge weights. We particularly focused on differences among edge weights, node strengths, and node EI.

#### 2.3.5 Network comparison

The NetworkComparisonTest (NCT) package was applied to analyze the differences in network analysis outcomes among epilepsy patients with varying seizure frequencies. Specifically, patients were categorized into a high-frequency group (seizures occurring daily, weekly, monthly, or every few months) and a low-frequency group. The comprehensive comparison capabilities of NCT allowed for the quantification of statistically significant discrepancies in the overall network layouts. Combined with a permutation tests (*n* = 1,000), the stability of network structures, the consistency of overall connection strength, and the uniformity of individual edge strengths were assessed. Network structure stability referred to the maximum difference in edge pairings between two networks; consistency of overall connection strength pertained to the disparity in the sum of all connections’ weights between networks; and uniformity in edge strength highlighted the variations in specific connection weights between the networks.

## 3 Results

### 3.1 Participant characteristics and the results of the one-way ANOVA

The average age of participants was 31.24 ± 12.17 years, ranging from 18 to 73, with 45.81% being female. Most surveyed epilepsy patients were employed, lived in urban areas, single, held associate degrees, had no family history of epilepsy, experienced generalized seizures, and had not had seizures in the past year. According to the results of the one-way ANOVA, differences in epilepsy-related fear scores among patients were statistically significant across variables such as gender, educational level, average household income (in RMB/month), place of residence, years since epilepsy diagnosis, major type of epilepsy, and Medication status of anti-seizure drugs. Additional details are available in Table 1. Table 2 lists the means and standard deviations for all items.

**Table 1:** Demographic characteristics of patients with epilepsy.

Variables		Number/N (%)	Mean ± SD	*F* value	*p*-value
Gender	Male	330 (54.19)	74.639 ± 15.819	4.389	0.037*
	Female	279 (45.81)	77.330 ± 15.756		
Age	18–25	231 (37.93)	76.065 ± 15.277	1.691	0.150
	26–35	220 (36.12)	76.841 ± 16.837		
	36–45	82 (13.46)	76.659 ± 12.230		
	46–55	34 (5.58)	70.676 ± 14.524		
	>55	42 (6.90)	72.405 ± 19.697		
Educational level	Elementary school and below	34 (5.58)	74.971 ± 21.675	2.561	0.026*
	Junior high school	117 (19.21)	75.735 ± 15.180		
	High school or vocational school	120 (19.70)	79.358 ± 14.703		
	Associate degree	168 (27.59)	76.708 ± 15.010		
	Bachelor’s degree	151 (24.79)	72.980 ± 16.692		
	Graduate degree	19 (3.12)	71.895 ± 10.257		
Marital status	Unmarried	347 (56.98)	75.905 ± 15.981	1.825	0.141
	Married	240 (39.41)	75.442 ± 15.795		
	Divorced	17 (2.8)	83.588 ± 12.674		
	Widowed	5 (0.82)	68.000 ± 11.247		
Average household income (in RMB/month)	≤2000	53 (8.70)	81.453 ± 13.999	5.83	0.000***
	2000–5,000 (including 5,000)	166 (27.26)	79.066 ± 17.271		
	5,000–10,000 (including 10,000)	218 (35.80)	74.472 ± 15.255		
	10,000–20,000 (including 20,000)	108 (17.73)	73.889 ± 13.274		
	>20,000	64 (10.51)	71.078 ± 16.906		
Place of residence	Urban area	352 (57.80)	73.940 ± 15.605	12.65	0.000***
	Rural area	257 (42.20)	78.518 ± 15.793		
Family history of epilepsy	Yes	35 (5.75)	76.886 ± 15.243	0.142	0.867
	No	525 (86.21)	75.884 ± 15.474		
	Unclear	49 (8.05)	75.020 ± 19.897		
Years since epilepsy diagnosis	0.5–1	67 (11.00)	70.154 ± 15.421	2.45	0.045*
	2–5	180 (29.56)	75.579 ± 16.490		
	6–10	136 (22.33)	76.897 ± 16.375		
	11–20	149 (24.47)	76.745 ± 15.451		
	>20	71 (11.66)	77.091 ± 15.676		
Major type of epilepsy	Focal aware seizures	87 (14.29)	73.218 ± 17.747	3.064	0.016*
	Focal impaired awareness seizures	76 (12.48)	77.408 ± 13.362		
	Generalized seizures	224 (36.78)	78.339 ± 14.207		
	Unclear/not classifiable	74 (12.15)	73.595 ± 17.921		
	Unknown	148 (24.30)	74.047 ± 16.593		
Frequency of epileptic seizures	Daily seizures	34 (5.58)	79.882 ± 17.841	1.921	0.075
	Weekly seizures	46 (7.55)	78.391 ± 19.064		
	Monthly seizures	117 (19.21)	77.564 ± 12.018		
	Yearly seizures	74 (12.15)	75.203 ± 12.089		
	Seizures every few months	81 (13.30)	77.432 ± 16.173		
	Seizures every few years	31 (5.09)	77.097 ± 16.304		
	No seizures in the past year	226 (37.11)	73.372 ± 17.178		
Medication status of anti-seizure drugs	Not taking medication	37 (6.08)	78.459 ± 11.594	3.859	0.004**
	1 type	265 (43.51)	73.396 ± 16.244		
	2 types	197 (32.35)	77.051 ± 16.965		
	3 types	86 (14.12)	77.547 ± 12.741		
	4 types and more	24 (3.94)	83.542 ± 13.072		
Last seizure	Within 1 day	34 (5.58)	78.529 ± 16.950	2.169	0.071
	within 1 week	60 (9.85)	79.800 ± 16.943		
	within 1 month	113 (18.56)	75.965 ± 13.988		
	within 1 year	187 (30.71)	76.460 ± 14.765		
	more than 1 year	215 (35.30)	73.795 ± 16.938		

*** indicates *p* < 0.0005, ** indicates *p* < 0.005, and * indicates *p* < 0.05.

**Table 2:** Statistical results of the mean and standard deviation of items (*N* = 609).

Item	Mean	SD
F1 I’m scared to suffocate during a seizure	2.46	0.81
F2 I’m afraid to get injured during a seizure	2.69	0.78
F3 I’m afraid to get urinary and fecal incontinence during seizures	2.57	0.85
F4 I’m afraid to experience painful things (like biting my tongue and forgetfulness) during a seizure	2.8	0.82
F5 I’m afraid to have a seizure at an unpropitious time or place (while swimming, bathing, crossing the street, in the party, driving, during recreation and while meeting someone important)	3.09	0.82
F6 I’m afraid to die during a seizure	2.57	0.85
F7 I’m afraid to have a seizure in front of others and they will notice my illness	2.98	0.83
F8 I’m afraid to be harassed by others during a seizure	2.67	0.81
F9 I’m afraid to have a seizure during sex or flirting	2.63	0.83
F10 I’m afraid others will make fun of me during the seizure	2.87	0.86
F11 I’m afraid my disease will be transferred to my child	3.17	0.85
F12 I’m afraid that taking drugs during pregnancy will cause physical problems in my fetus	2.92	0.97
F13 I’m afraid of not controlling for potential harmful conditions during a seizure	3.02	0.79
F14 In times of insomnia and fatigue, I have a fear of seizures	3	0.77
F15 I’m afraid that others will contact me for compassion or keep their relationship with me	2.41	0.87
F16 I’m afraid that my sexual performance will be diminished due to taking antiepileptic drugs	2.53	0.88
F17 I’m afraid the number and duration of seizure attacks will worsen in the future	3.05	0.81
F18 I’m afraid of being discriminated in relationships with others	2.88	0.86
F19 I’m afraid to get brain damage over time due to many seizures	3.2	0.76
F20 I am afraid in the long run antiepileptic drugs will damage my body	3.12	0.76
F21 I’m afraid seizures will accompany me all of my life	3.19	0.78
F22 I’m afraid that others will feel that I am different from them and reject me	2.83	0.86
F23 I’m afraid I will never be able to control or manage my illness in the future	3.01	0.81
F24 I’m afraid to depend on others for my illness in the future	2.99	0.83
F25 I’m afraid of losing my friends/colleagues because of their fear of contagiousness of my disease and they will not let me engage in their gathering	2.39	0.9
F26 I’m afraid that my family members or friends will treat me compassionately over time	2.41	0.88
F27 I’m afraid in the long run my family will lose hope of curing my illness	2.42	0.96

### 3.2 The results for reliability and validity

#### 3.2.1 Exploratory network analysis

**Figure 1:**
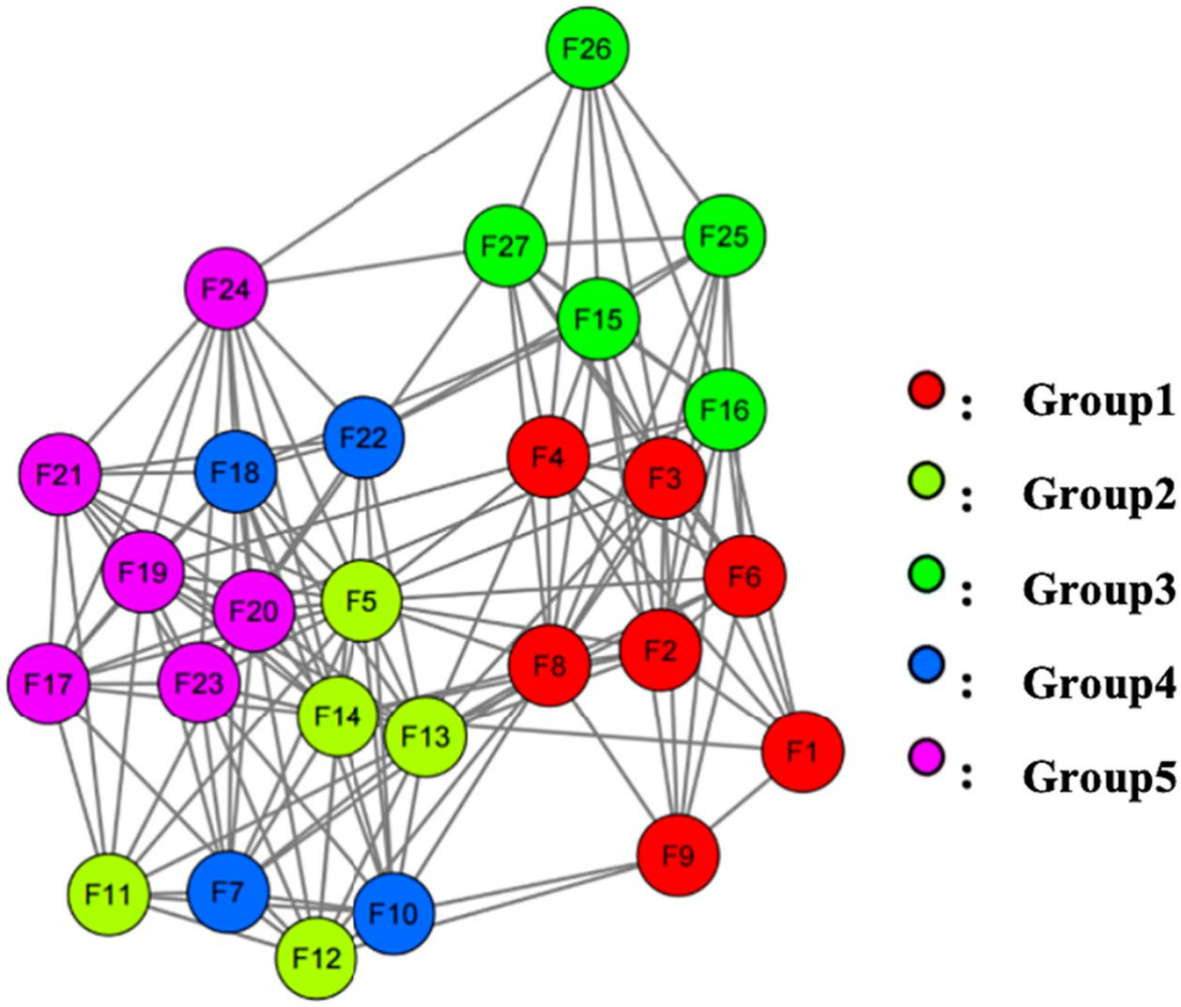
Exploratory graph analysis indicates a 5-group structure for the D-RFS.

The KMO value was 0.96, and the Bartlett’s test of sphericity yielded a significant result (*χ*^2^ = 13911.63, df = 351, *p* < 0.001), indicating that the data were very suitable for EFA. The 27 items of the D-RFS formed 5 item groups. As can be seen from Figure 1, Group 1 is composed of F1, F2, F3, F4, F6, F7, F8, and F9; Group 2 consists of F5, F11, F12, F13, and F14; Group 3 is made up of F15, F16, F25, F26, and F27; Group 4 includes F7, F10, F18, and F23; Group 5 is constituted by F17, F19, F20, F21, F22, and F24.

#### 3.2.2 Confirmatory factor analysis

In the CFA analysis of the 5-group model derived from the D-RFS network, the null hypothesis of exact-fit was rejected (*χ*^2^ (289) = 1463.984, *p* < 0.001). A detailed review of the comparative fit indices revealed the following: IFI (0.912), CFI (0.912), TLI (0.901), RNI (0.912), and NFI (0.892), with SRMR (0.052) and R-RMSEA (0.082). After applying modifications suggested by indices, such as linking residuals between item pairs (7 and 10; 11 and 12), there was a reduction in *χ*^2^. Subsequent adjustments led to an enhanced model fit with *χ*^2^ (287) reduced to 1222.458, *p* < 0.001, and improved indices: IFI (0.930), CFI (0.930), TLI (0.920), RNI (0.930), NFI (0.910), a refined SRMR (0.051), and a lower R-RMSEA (0.073). The AVE values, namely 0.606, 0.535, 0.730, 0.723, and 0.700, underscored robust convergent validity.

When we compare the model fit indices adjusted by network exploratory analysis with those adjusted by traditional exploratory analysis methods (Table 3), we observe that the fit indices derived from network exploratory analysis demonstrate superior performance compared to those obtained through traditional methods.

**Table 3:** Comparative assessment of model fit indices: network exploratory analysis versus traditional methods.

	*χ*^2^/df	SRMR	RMSEA	IFI	CFI	TLI	RNI	NFI
Model 1	4.260	0.051	0.073	0.930	0.930	0.920	0.930	0.910
Model 2	3.490	0.061	0.091	0.884	0.883	0.871	0.883	0.844

Model 1 represents the five-factor model derived from network exploratory analysis, while Model 2 refers to the three-factor model obtained through traditional methods.

#### 3.2.3 Reliability

The study demonstrated outstanding overall reliability with a Cronbach’s *α* of 0.960. Strong reliability was confirmed across each group, with alpha coefficients ranging from 0.864 to 0.933. The results also indicated robust reliability, evidenced by a Macdonald’s Omega (*ω*) coefficient of 0.946. Detailed statistics are presented in Table 4.

**Table 4:** Reliability.

	Cronbach’s *α*	*ω* coefficient
Group1	0.914	0.915
Group2	0.864	0.819
Group3	0.912	0.915
Group4	0.919	0.889
Group5	0.933	0.933
Total	0.960	0.946

### 3.3 Network estimation

**Figure 2:**
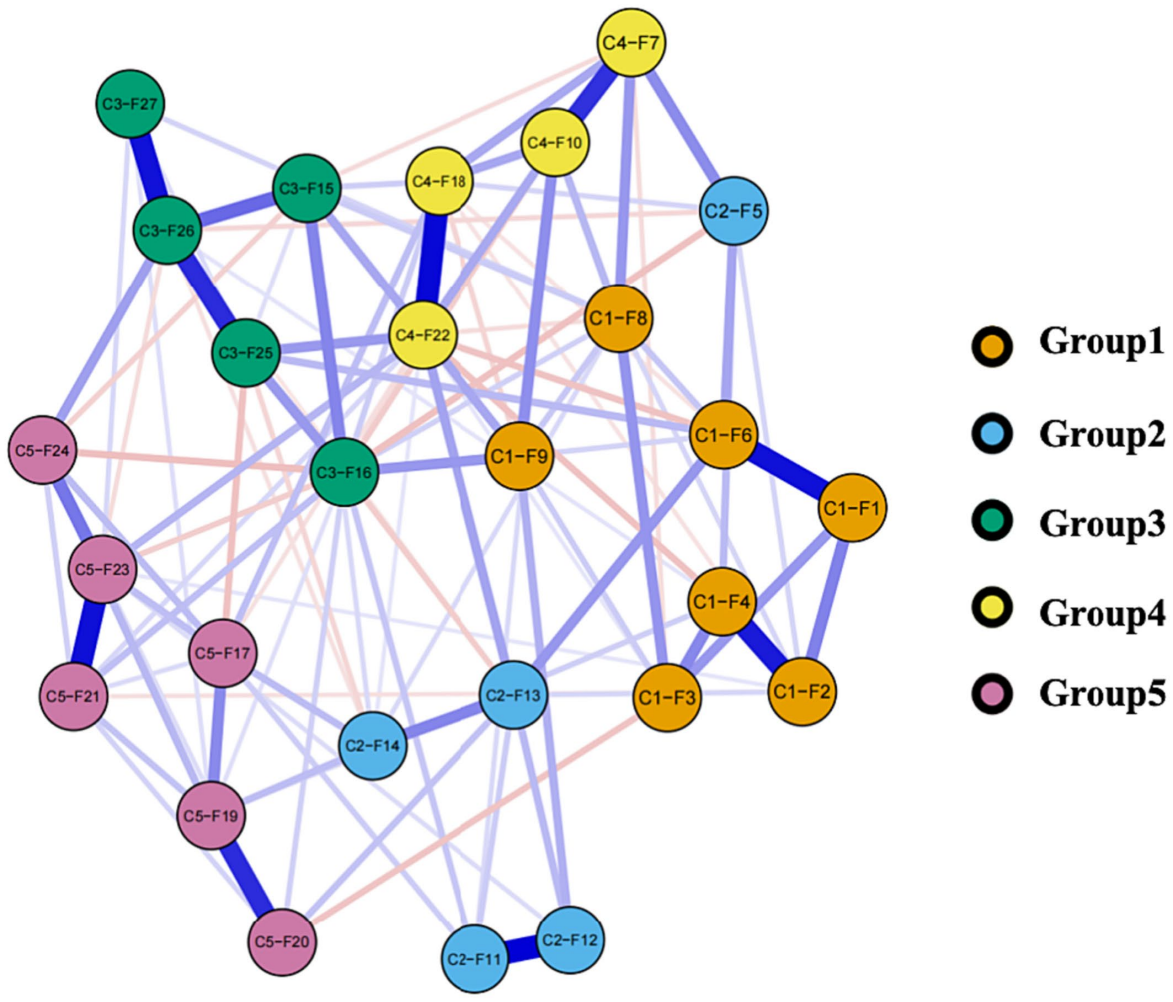
Network of disease-related fear symptoms. In this diagram, nodes with stronger correlations are closer to each other. The thickness of an edge indicates the strength of the correlation. Blue lines indicate positive associations, while red lines indicate negative associations.

The cross-sectional network of disease-related fear symptoms in patients with epilepsy, estimated using the EBICglasso model, is presented in Figure 2. Each node represents an individual questionnaire item; therefore, all nodes are interconnected, and there are no isolated nodes.

#### 3.3.1 The characteristics of edges

Logically, there could have been a maximum of 378 possible edges in the network, each representing a potential relationship between different items. However, in the present research, only 220 edges were deemed significant due to their absolute weight being greater than zero, as illustrated in Figure 1. Furthermore, there are 71 edges (ranging from −0.128 to 0.232) among the five groups in the network, with positive edges accounting for the majority of the inter-group edges (64.79%) as shown in Table 5. Weights are a representation of the correlation between two different items or variables. Where a positive weight suggests a positive correlation, negative weights indicate a contrary relationship. The intensity of this correlation is precisely embodied by the size of the weight’s absolute value (34). This becomes clear in an example: When symptoms of C2-F5 appear, the emergence of C4-F7 symptoms is concurrent. This observation implies that individuals manifesting C2-F5 symptoms are highly likely to also exhibit C4-F7 symptoms. However, in the realm of negative weights, a reverse situation is observed. If symptoms of C1-F3 are conspicuous, C4-F20 symptoms generally become less evident. Thus, for the same individual, the simultaneous occurrence of symptoms C1-F3 and C4-F20 is uncommon.

**Table 5:** The most robust edges.

Group	Group	The most robust edges	Weight
Group1	Group2	C1-F6——C2-F13	0.209
Group1	Group3	C1-F9——C3-F16	0.210
Group1	Group4	C1-F9——C4-F10	0.227
Group1	Group5	C1-F3——C4-F20	−0.118
Group2	Group3	C2-F5——C3-F16	−0.124
Group2	Group4	C2-F5——C4-F7	0.232
Group2	Group5	C2-F14——C5-F17	0.133
Group3	Group4	C3-F25——C4-F22	0.206
Group3	Group5	C3-F16——C4-F24	−0.128
Group4	Group5	C4-F18——C5-F17	0.129

#### 3.3.2 Results of node centrality indices

**Figure 3:**
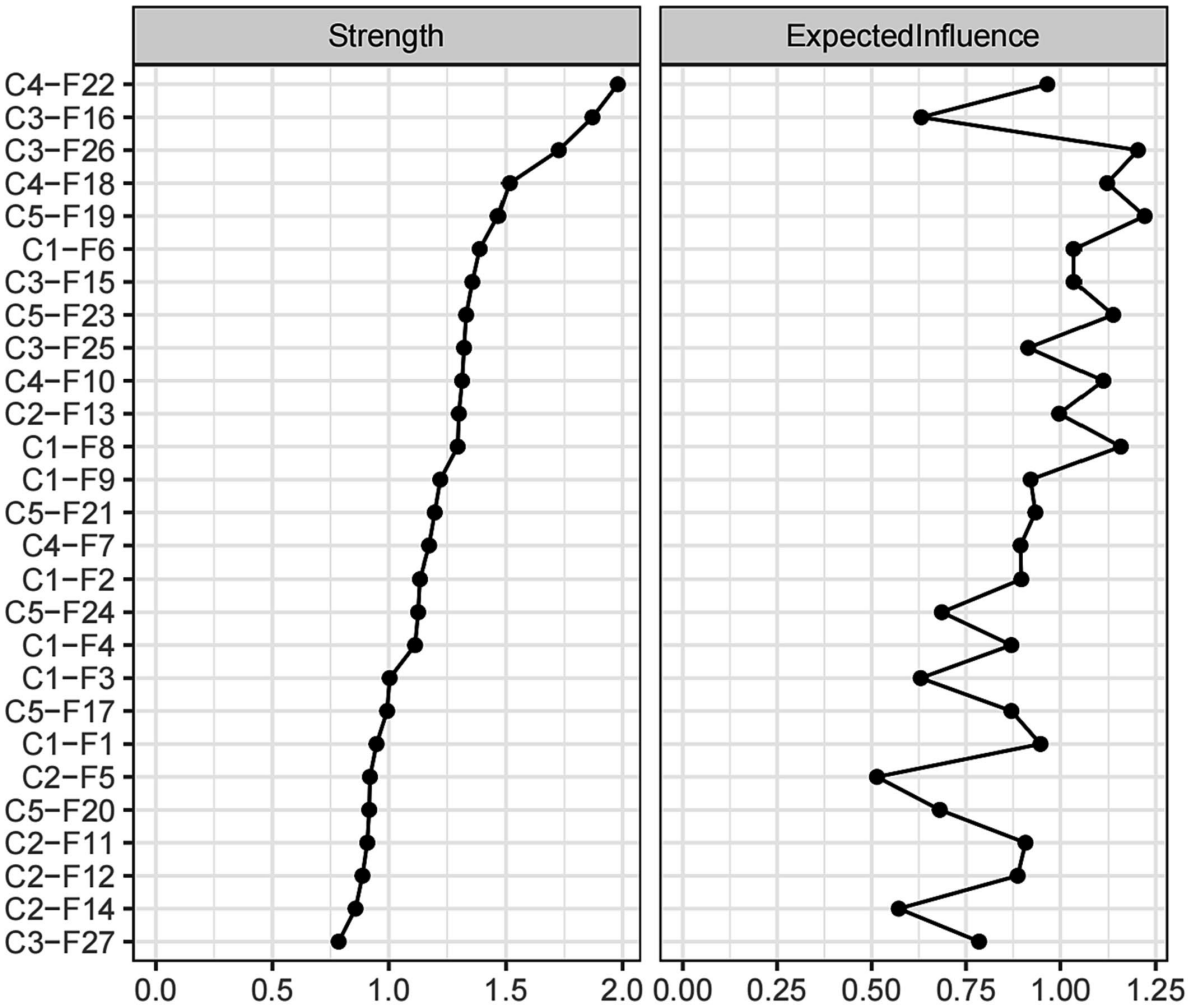
Centrality indices of disease-related fear symptoms.

Figure 3 shows the network centrality indices. C4-F22 (strength = 1.979, EI = 0.965) was the highest node strength (with more direct connections with other nodes), followed by C3-F16 (strength = 1.870, EI = 0.631). C5-F19 (strength = 1.467, EI = 1.221) showed the highest expected influence, followed by C3-F26 (strength = 1.203, EI = 1.726). Notably, C3-F27 (strength = 0.785, EI = 0.785) was the lowest node strength in the network, and C2-F5 (strength = 0.919, EI = 0.514) was the lowest node expected influence. In essence, C4-F22’s prominent node strength suggests its significant role in the network owing to its multitude of direct connections, while C5-F19, due to its supreme expected influence, is considered to exert the most substantial impact on other network nodes.

#### 3.3.3 Results of bridge centrality indices

**Figure 4:**
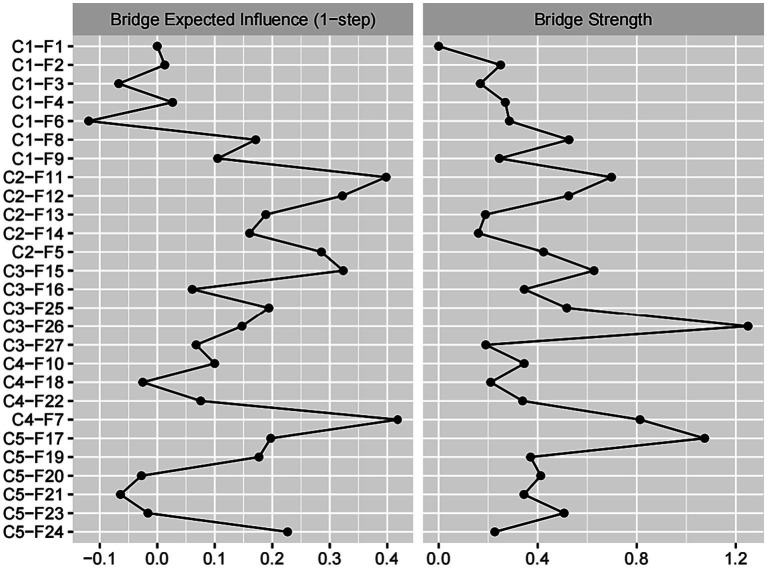
Bridge centrality indices of disease-related fear symptoms.

Bridge centrality is depicted in Figure 4. An examination of the network (as shown in Figure 2) reveals that the five groups have each established a nearly stable structure with interconnections among them. Specifically, the top 20th percentile of nodes according to the BEI includes C4-F7 (BEI = 0.419, bridge strength = 0.813), C2-F11 (BEI = 0.398, bridge strength = 0.698), C2-F12 (BEI = 0.322, bridge strength = 0.525), C3-F15 (BEI = 0.323, bridge strength = 0.628), and C2-F5 (BEI = 0.285, bridge strength = 0.424). Furthermore, nodes in the top 20th percentile for bridge strength are C3-F26 (BEI = 0.147, bridge strength = 1.245), C5-F17 (BEI = 0.197, bridge strength = 1.074), C4-F7 (BEI = 0.419, bridge strength = 0.813), C3-F15 (BEI = 0.323, bridge strength = 0.627), and C2-F11 (BEI = 0.398, bridge strength = 0.698). It can be clearly observed that in comparison to nodes in other groups, nodes within group 1 generally exhibit lower BEI and strength indices. In addition, C4-F7 plays a crucial role in group interactions due to its highest BEI, while C3-F26 becomes a key node in the interaction between different groups because of its highest bridge strength.

### 3.4 Network accuracy and stability

In the network of disease-related fear symptoms, the 95% confidence interval of edge weights was narrow, indicating acceptable accuracy of the edge weights (Supplementary Figure S1). As illustrated in Supplementary Figure S3, with the decrease in subsample size, the average correlation of strength and EI indices between the original sample and subsamples also decreased. In this context, the CS coefficient for strength was 0.438, surpassing the threshold of 0.25, signifying acceptable stability. Moreover, the CS coefficient for EI was 0.594, exceeding the threshold of 0.5, demonstrating a satisfactory level of stability. Simultaneously, significance tests for differences in EI indicated that C5-F19 emerged as the most influential node, displaying a significantly greater impact compared to the other nodes (Supplementary Figure S3). In the analysis of edge weight differences, the results revealed that the edge weight between C2-F5 and C4-F7 was the largest among inter-group edges (Supplementary Figure S4).

### 3.5 Network comparison

**Figure 5:**
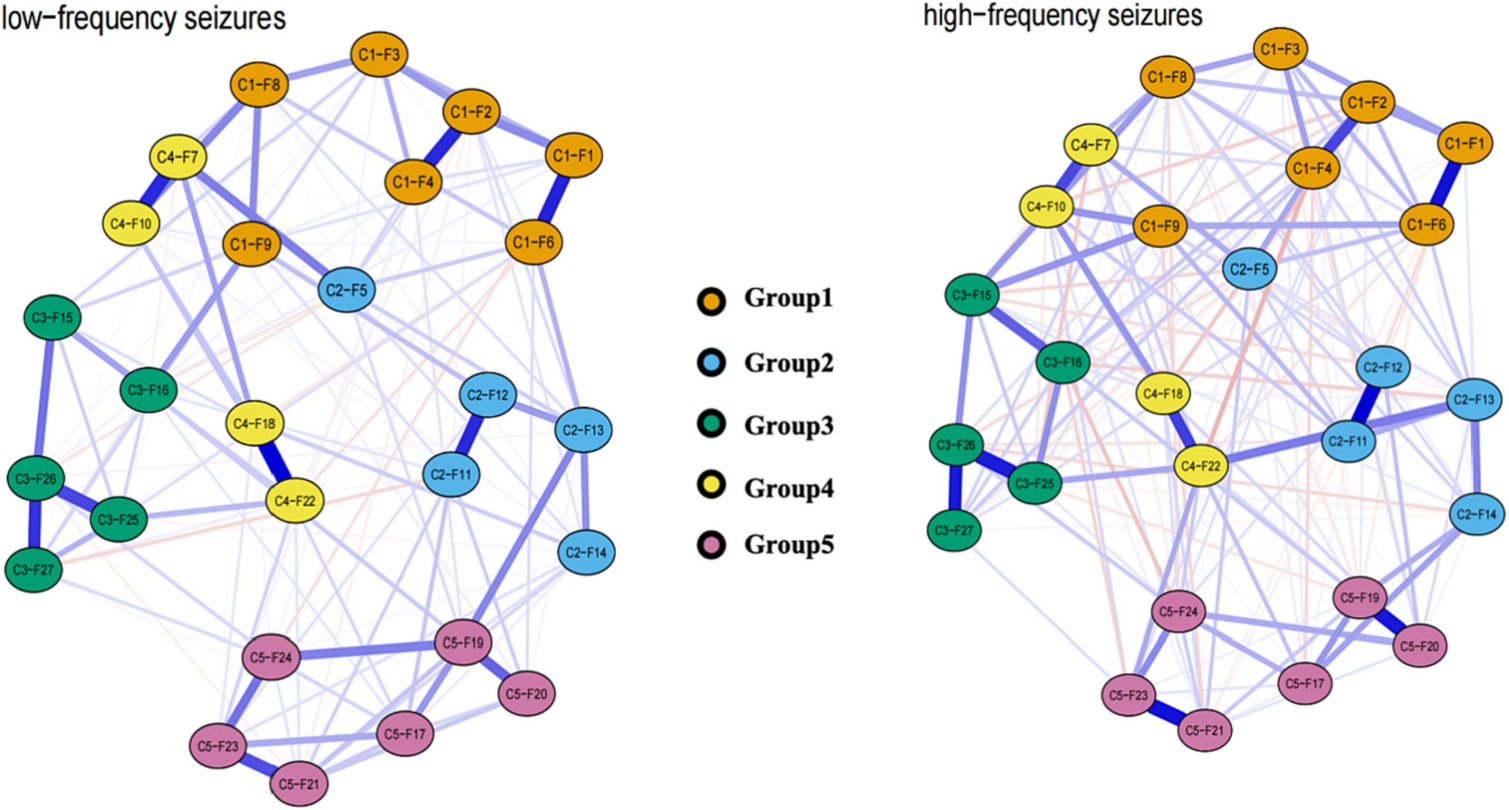
Estimated network model between low-frequency seizure group and the high-frequency seizure group.

During the comparison of network models across varying seizure frequencies (Figure 5), we observed no significant changes in global network strength (*p* = 0.278), nor did we find significant differences with the Network Invariance Test (*p* = 0.495). The edge invariance test results indicated that most edges did not show statistical significance (*p* > 0.05), however, 25 edges showed *p* values below 0.05 (Supplementary Table S1) such as the edges between C1-F2 and C1-F3, C1-F8 and C4-F10, C2-F13 and C5-F19, as well as C4-F10 and C5-F21. The majority of nodes had p values above 0.05, indicating no significant differences in centrality between networks. However, nodes C5-F17 and C5-F19 were exceptions, with *p* values of 0.0495 and 0.0297, respectively, indicating significant differences in these nodes across the networks tested.

## 4 Discussion

This study is the first to investigate the phenomenon of disease-related fear in patients with epilepsy by means of network analysis. Departing from conventional methodologies, network theory furnishes us with a novel approach to construct psychological frameworks. It posits that the core of psychological disorders lies in the intricate interplay between symptoms (39). These interactions weave together to form a complex network system, complete with feedback loops among the symptoms. Given this dynamic state of self-maintenance, spontaneous recovery of psychological disorders becomes challenging (39, 40). Consequently, within this research, we conceptualize disease-related fear in patients with epilepsy as an interactive system. This system amalgamates diverse manifestations of disease-related fear, unveiling a fresh perspective for comprehending and depicting these phenomena. To be more precise, through this approach, we attain an enhanced capability to pinpoint pivotal factors within the network, subsequently employing visual maps to comprehensively unveil the interrelationships among various fear representations. This holistic visual representation greatly aids in delving deeper into the intricacies and inherent connections underlying the disease-related fear in patients with epilepsy. This research generally aims to identify and confirm the factor structure through network analysis. The goal is to understand the fundamental mechanisms and internal connections of disease-related fears among epilepsy patients and compare these findings with the results from traditional exploratory factor analysis. Moreover, centrality indicators are applied to measure key elements within the network, promising to provide insights for the design of targeted new intervention strategies. The network model maintains an acceptable level of stability and accuracy, thus supporting the credibility of the research conclusions. Comparative analysis of various network models is carried out under different seizure frequencies to ensure result accuracy.

Our research presents a five-group structure for the D-RFS based on exploratory network analysis. The new 5-group structure found in the instrument signifies a shift in the D-RFS’ structure in the Chinese culture. The initial factor “Fear of poor epilepsy management” has been split into three separate groups: Group 2 (containing items F5, F11, F12, F13, F14), Group 4 (containing items F7, F10, F18, F22), and Group 5 (containing items F17, F19, F20, F21, F23, F24). This has resolved inconsistencies with question placement, improving item allocation. These groups have been named as “Fear of Seizure Consequences,” “Fear of Poor Epilepsy Management,” “Fear of Social Restrictions,” “Fear of Public Perception”, and “Fear of Illness Progression.” The five-group structure further proved through a confirmatory factor analysis to accurately reflect and explain data structures. Ultimately, the scores exhibit outstanding consistency in measurements.

The research results reveal that fear symptoms within the four groups form a closely intertwined cluster. Only Dimension 2’s F5 is closely related to specific items in Dimension 1 and Dimension 4. This suggests the potential involvement of similar fear symptom constructs or even possible causal relationships (39). For example, between C2-F5 “I’m afraid to have a seizure at an unpropitious time or place (while swimming, bathing, crossing the street, in the party, driving, during recreation and while meeting someone important)” and C1-F6 “I’m afraid to die during a seizure “, the concern about being unable to control potential harm could exacerbate the fear of dying during an epileptic seizure, aligning with Cramer’s findings (40).The most significant connections include C1-F9 “I’m afraid to have a seizure during sex or flirting” and C4-F10 “I’m afraid others will make fun of me during the seizure.” Both reflect an individual’s inner experiences and concerns in social and intimate situations. Also, the connections between C4-F7 “I’m afraid to have a seizure in front of others and they will notice my illness” and C1-F8 “I’m afraid to be harassed by others during a seizure” are notably robust. The fear of epilepsy patients being noticed during a seizure extends beyond the fear of being ridiculed, potentially including concerns about post-seizure humiliation and violation (7, 41).

Notably, nodes in Groups 3 and 4 exhibit a high degree of node centrality. Moreover, multiple entries within these two groups are correlated, and nodes within these groups interact with each other, forming a stable network structure. This structure tends to make Chinese epilepsy patients more susceptible to entering a cycle of fear related to their condition. We understand that China exhibited diverse local beliefs across various regions, where epilepsy is often perceived as a psychiatric disorder rather than a neurological condition (42, 43). Such attribution carries negative connotations, resulting in societal constraints and discrimination against individuals with epilepsy (44). Moreover, Chinese individuals with epilepsy often experience heightened feelings of shame (45), further intensifying the social limitations (46). Taking these factors into consideration, our research findings seem to suggest that the “Fear of Social Restrictions” and “Fear of Public Perception” play a significant role in the fear symptoms of Chinese epilepsy patients. Given that core symptoms exert the most influence over the entire network (47), we recommended focusing interventions on these core symptoms. Therefore, for Chinese patients with epilepsy, especially the support and encouragement of family members or friends are crucial. This can effectively assist them in overcoming the negative impact of fear and consequently enhance their quality of life.

Bridge strength centrality gauges the cumulative strength of connections between a node and all nodes in other groups, thereby reflecting the node’s significance in bridging different groups. Consequently, bridge strength more precisely illuminates the inter-group relationships, offering more effective targets for interventions. According to network and bridge centrality metrics, it can be observed that the connections between different groups tend to be tight. The various fear groups are not existing in isolation, but rather they influence and reinforce each other, thereby imposing greater psychological stress and challenges on epilepsy patients (48). Therefore, any intervention aiming at this network structure should consider all the relevant fear dimensions comprehensively to achieve optimal therapeutic outcomes. For instance, healthcare professionals could disseminate scientific knowledge about epilepsy to patients via diverse channels, including online platforms. They could elucidate risks in various scenarios and provide practical behavioral recommendations to help patients better navigate diverse situations in their daily lives (49). Additionally, mindfulness-based intervention techniques can aid patients in concentrating on the present circumstances, thereby lessening anxiety stemming from prospective uncertainties and fortifying mental resilience to alleviate fear (50, 51). Beyond individual-level interventions, societal support is of paramount importance, particularly in rural areas of China where epilepsy stigmatization is more pronounced (42). Consequently, endeavors to establish affirmative awareness about epilepsy in society are pivotal for fostering comprehension and acceptance of epilepsy patients (52). Furthermore, the backing of family and friends significantly impacted the psychological well-being of epilepsy patients, as their emotional support and encouragement played a pivotal role in patients’ recovery and heightened self-assurance (53).

In this research, we observe significant discrepancies in the edge weights of seizure frequency, despite the unaltered network structure itself. We discover that the networks with more frequent seizures have a higher volume of connections compared to the ones with less frequent episodes. This finding suggests that with an increased seizure frequency, the correlations between elements or symptoms within the network become tighter, consequently strengthening their mutual influence. Therefore, while tackling patients with frequent seizures, we need an intensive observation for any symptom changes, an active search for potential triggering factors, and an urgent need for early intervention (6, 48). Moreover, we might need to implement various treatment methods simultaneously, aiming to control the seizure frequency and address corresponding symptoms.

There are several limitations that need to be acknowledged in this study. Firstly, the study design does not allow us to determine whether the most central symptom is the cause of activating other symptoms or is instead a consequence of other symptoms. Given that our analysis was based on cross-sectional data, the direction of edges remains undetermined. As a result, the relationships discussed in this paper cannot be confidently interpreted as causal; establishing causality would require longitudinal data analysis. Secondly, the study relied on self-report questionnaires, which could introduce subjectivity into the results. Additionally, certain influential factors, including social support, family dynamics, disease severity, frequency of epileptic seizures, and the status of anti-seizure medication, were not considered in this study, but these factors were not the focus of the current study. Furthermore, the study does not incorporate other psychological measurement scales such as depression and anxiety, potentially impacting the stability of the “fear network” under the influences of these psychological constructs. Therefore, there may be some deficiencies in confirming the construct validity.

To address the uncertainty regarding the causality between core symptoms and other symptoms, future longitudinal studies could be conducted. Further longitudinal research could help us understand the directionality of these bridge pathways. Furthermore, expanding the scope of the study to explore the relationship between disease-related fear in epilepsy patients and psychological disturbances such as depression and anxiety, as well as investigating the association between disease-related fear items in epilepsy patients and potential psychological and social consequences, would provide a more comprehensive understanding of underlying mechanisms and potential intervention targets.

## 5 Conclusion

In summary, the visual network structure paints a detailed roadmap of the connections between disease-related fear symptoms in patients with epilepsy. Through exploratory network analysis, we further confirmed more appropriate group dimensions and established a five-dimension structure. Interestingly, the discovery of this five-dimensional model does not contradict the three-dimensional structure that was previously derived from traditional exploratory factor analysis. In fact, these factor structures have been deciphered in a more comprehensive and in-depth manner, and our analytical findings are both solid and credible. From the perspective of network analysis, the nodes of “Fear of Social Restrictions” and “Fear of Public Perception” have a high node centrality, making Chinese epilepsy patients more prone to fall into a cycle of disease-related fear. Further network centrality analysis revealed that nodes between dimensions show strong bridge centrality, indicating a close connection among groups. Additionally, this research is the first application of network analysis to explore the relationships between disease-related fear symptoms among epilepsy patients, with the aim to provide a reliable reference for psychological intervention practices.

## Data availability statement

The original contributions presented in the study are included in the article/Supplementary material, further inquiries can be directed to the corresponding author.

## Ethics statement

The studies involving humans were approved by the ethics committee of the Second Affiliated Hospital, Zhejiang University School of Medicine (SAHZU, number: 2023-0062). The studies were conducted in accordance with the local legislation and institutional requirements. The participants provided their written informed consent to participate in this study.

## Author contributions

XY: Formal analysis, Investigation, Methodology, Software, Validation, Visualization, Writing – original draft, Writing – review & editing. SN: Data curation, Methodology, Software, Visualization, Writing – original draft, Writing – review & editing. QY: Investigation, Software, Writing – review & editing. YX: Investigation, Writing – review & editing. XF: Supervision, Writing – review & editing.

## References

[1] Gandy M Michaelis R Acraman J Donald KA Fitzpatrick M WC LF. Integrated psychological care services within seizure settings: key components and implementation factors among example services in four ILAE regions: a report by the ILAE psychiatry commission. Epilepsia. (2023):e1766. doi: 10.1111/epi.17647 37227085

[2] Stirling RE Cook MJ Grayden DB Karoly PJ. Seizure forecasting and cyclic control of seizures. Epilepsia. (2021) 62:S2. doi: 10.1111/epi.16541, PMID: 32712968

[3] Choy M Dadgar-Kiani E Cron GO Duffy BA Schmid F Edelman BJ. Repeated hippocampal seizures lead to brain-wide reorganization of circuits and seizure propagation pathways. Neuron. (2022) 110:221-36e4. doi: 10.1016/j.neuron.2021.10.010 34706219 PMC10402913

[4] Cronin W Kwan P Foster E. Anxiety and depressive symptoms in adults with new-onset seizures: a scoping review. Epilepsia Open. (2023) 4:12766. doi: 10.1002/epi4.12766 PMC10472411 37247255

[5] Rauh R Schulze-Bonhage A Metternich B. Assessment of anxiety in patients with epilepsy: a literature review. Front Neurol. (2022) 13:836321. doi: 10.3389/fneur.2022.836321, PMID: 35547374 PMC9081800

[6] Tarrada A Aron O Vignal JP Ertan D Maillard L Hingray C. Anticipatory anxiety of seizures is associated with ictal emotional distress and amygdala onset seizures. Epilepsia. (2022) 63:1130. doi: 10.1111/epi.17215 35263805

[7] Shamsalinia A Moradi M Farahani MA Masoudi R Ghadimi R Rad RE. Designing and psychometric evaluation of disease-related fear scale (D-RFS) in adults with epilepsy: a sequential exploratory mixed methods design. Epilepsy Behav. (2020) 110:107169. doi: 10.1016/j.yebeh.2020.107169 32504981

[8] Chen J Li Q Tong X Su M Wang C Zhou D. Epilepsy-related concerns among patients with epilepsy in West China. Epilepsy Behav. (2018) 82:128. doi: 10.1016/j.yebeh.2018.02.014, PMID: 29625362

[9] Weiss A Canetti L David SB Reuveni I Ekstein D. Seizure phobia: a distinct psychiatric disorder among people with epilepsy. Seizure. (2022) 95:26. doi: 10.1016/j.seizure.2021.12.009, PMID: 34974230

[10] Wei Z Wang X Ren L Liu C Liu C Cao M. Using machine learning approach to predict depression and anxiety among patients with epilepsy in China: a cross-sectional study. J Affect Disord. (2023) 336:1. doi: 10.1016/j.jad.2023.05.043, PMID: 37209912

[11] Richter D Clever K Mehnert-Theuerkauf A Schönfelder A. Fear of recurrence in young adult cancer patients-a network analysis. Cancers. (2022) 14:2092. doi: 10.3390/cancers14092092, PMID: 35565220 PMC9105535

[12] Luo X Li W Chen Y Sun H Humphris G Liu T. Fear of recurrence in Chinese cancer patients: prevalence, correlates, and network analysis. Psychiatry. (2022) 13:803543. doi: 10.3389/fpsyt.2022.803543, PMID: 35197876 PMC8859333

[13] Galderisi S Rucci P Kirkpatrick B Mucci A Gibertoni D Rocca P. Interplay among psychopathologic variables, personal resources, context-related factors, and real-life functioning in individuals with schizophrenia: a network analysis. JAMA Psychiatry. (2018) 75:396–404. doi: 10.1001/jamapsychiatry.2017.4607, PMID: Retraction in: JAMA Psychiatry. 2018 Jul 1;75(7):754-755 29450447 PMC5875306

[14] Letouche S Wille B. Connecting the dots: exploring psychological network analysis as a tool for analyzing organizational survey data. Front Psychol. (2022) 13:838093. doi: 10.3389/fpsyg.2022.838093, PMID: 35592177 PMC9110883

[15] Epskamp S Waldorp LJ Mõttus R Borsboom D. The Gaussian graphical model in cross-sectional and time-series data. Multivariate Behav Res. (2018) 53:453–80. doi: 10.1080/00273171.2018.1454823, PMID: 29658809

[16] Hofmann SG Curtiss J Mcnally RJ. A complex network perspective on clinical science. Perspect Psychol Sci. (2016) 11:597–605. doi: 10.1177/1745691616639283 27694457 PMC5119747

[17] Park M Seo D Kim JG Lee G McReynolds L Amsel L. Identification and comparison of pandemic-to-symptom networks of South Korea and the United States. Front Psych. (2023) 14:1161200. doi: 10.3389/fpsyt.2023.1161200, PMID: 37426108 PMC10328092

[18] Cai H Bai W Yue Y Zhang L Mi WF Li YC. Mapping network connectivity between internet addiction and residual depressive symptoms in patients with depression. Front Psych. (2022) 13. doi: 10.3389/fpsyt.2022.997593, PMID: 36353572 PMC9638086

[19] Yang T He Y He C Yang Y Wu L Wei B. The relationship between anxiety and internet gaming disorder in children during COVID-19 lockdown: a network analysis. Front Psych. (2023) 14:144413. doi: 10.3389/fpsyt.2023.1144413, PMID: 37265552 PMC10229880

[20] Gong Y Guo Z Lu H Wang X Zhang Y Ren L. Network analysis of acute stress reaction in a sample of Chinese male military college students. Front Psych. (2023) 14:1082549. doi: 10.3389/fpsyt.2023.1082549, PMID: 37621968 PMC10444979

[21] Cascino G Marciello F D’Agostino G Toricco R Barone E Monteleone AM. Using network analysis to explore the association between eating disorders symptoms and aggressiveness in bulimia nervosa. Front Psych. (2022) 13:907620. doi: 10.3389/fpsyt.2022.907620, PMID: 36090364 PMC9451028

[22] Yin X Niu S Yu Q Xuan Y Chen L Feng X. Validity and reliability of the Chinese version of the disease-related fear scale in patients with epilepsy. Epilepsy Behav. (2023) 147:109404. doi: 10.1016/j.yebeh.2023.109404, PMID: 37683426

[23] Turan GB Özer Z Karman S. Turkish validity and reliability study of disease-related fear scale in patients with epilepsy. Epilepsy Behav. (2023) 138:109053. doi: 10.1016/j.yebeh.2022.109053 36543043

[24] Rauh R Domschke K Hirsch M Schulze-Bonhage A. Listening to anxiety in persons with epilepsy. Development of an integrative assessment model based on qualitative interviews. Epilepsy Behav. (2023) 145:109319. doi: 10.1016/j.yebeh.2023.109319, PMID: 37406558

[25] DeVon HA Block ME Moyle-Wright P Ernst DM Hayden SJ Lazzara DJ. A psychometric toolbox for testing validity and reliability. J Nurs Scholarsh. (2007) 39:155–64. doi: 10.1111/j.1547-5069.2007.00161.x, PMID: 17535316

[26] Brown TA. Confirmatory factor analysis for applied research. New York: Guilford publications (2015).

[27] Ab Hamid M R Sami W Sidek M H M. Discriminant validity assessment: use of Fornell & Larcker criterion versus HTMT criterion; proceedings of the journal of physics: Conference Series, (2017), IOP Publishing

[28] Mayers A. Introduction to statistics and SPSS in psychology. (2013)

[29] Ataya AF Adams S Mullings E Cooper RM Attwood AS Munafò MR. Internal reliability of measures of substance-related cognitive bias. Drug Alcohol Depend. (2012) 121:148–51. doi: 10.1016/j.drugalcdep.2011.08.023, PMID: Retraction in: Drug Alcohol Depend. 2012 Aug 1;124(3):189-90 21955365

[30] Epskamp S, Fried E I.Package ‘bootnet’.R package version, (2018)

[31] Epskamp S Cramer AO Waldorp LJ Schmittmann VD Borsboom D. Qgraph: network visualizations of relationships in psychometric data. J Stat Softw. (2012) 48:1–18. Retraction in: Drug Alcohol Depend. 2012 Aug 1;124(3):189-90. doi: 10.18637/jss.v048.i04

[32] Epskamp S Borsboom D Fried EI. Estimating psychological networks and their accuracy: a tutorial paper. Behav Res Methods. (2018) 50:195–212. doi: 10.3758/s13428-017-0862-1 28342071 PMC5809547

[33] Epskamp S Fried EI. A tutorial on regularized partial correlation networks. Psychol Methods. (2018) 23:617. doi: 10.1037/met0000167 29595293

[34] Newman M. E., The mathematics of networks, The new palgrave encyclopedia of economics, (2008), 2, 1–12. doi: 10.1057/978-1-349-95121-5_2565-1

[35] Bringmann LF Elmer T Epskamp S Krause RW Schoch D Wichers M. What do centrality measures measure in psychological networks? J Abnorm Psychol. (2019) 128:892–903. doi: 10.1037/abn0000446 31318245

[36] Mcnally RJ. Can network analysis transform psychopathology? Behav Res Ther. (2016) 86:95–104. doi: 10.1016/j.brat.2016.06.006 27424882

[37] Jones PJ Ma R Mcnally RJ. Bridge centrality: a network approach to understanding comorbidity. Multivariate Behav Res. (2021) 56:353–67. doi: 10.1080/00273171.2019.1614898 31179765

[38] Jones P Jones M P. Package ‘networktools’.Internet, cited 2018 June, Available at: https://cranrprojectorg/web/packages/networktools/networktoolspdf. (2017)

[39] Borsboom D. A network theory of mental disorders. World Psychiatry. (2017) 16:5–13. doi: 10.1002/wps.20375 28127906 PMC5269502

[40] Cramer AO van Borkulo CD Giltay EJ van der Maas HLJ Kendler KS Scheffer M. Major depression as a complex dynamic system. PLoS One. (2016) 11:e0167490. doi: 10.1371/journal.pone.0167490, PMID: 27930698 PMC5145163

[41] Stangl AL Earnshaw VA Logie CH Brakel W Simbayi LS Brrre I. The health stigma and discrimination framework: a global, crosscutting framework to inform research, intervention development, and policy on health-related stigmas. BMC Med. (2019) 17:e0167490. doi: 10.1186/s12916-019-1271-3 PMC6376797 30764826

[42] Ding D Zhou D Sander JW Wang W Li S Hong Z. Epilepsy in China: major progress in the past two decades. Lancet Neurol. (2021) 20:316–26. doi: 10.1016/S1474-4422(21)00023-5, PMID: Retraction in: Lancet Neurol. 2021 May;20(5):333-334 33743240

[43] Yue Z Ma C Lim KS Xiao B Wu Q Shu Y. Validation of the Chinese version of public attitudes toward epilepsy scale in mainland China. Epilepsy Behav. (2017) 72:150–5. doi: 10.1016/j.yebeh.2017.04.028, PMID: 28582727

[44] Mula M Kaufman KR. Double stigma in mental health: epilepsy and mental illness. BJPsych Open. (2020) 6:e72. doi: 10.1192/bjo.2020.58 32654672 PMC7443902

[45] Tang B Fu Y Liu B Yi Q. Self-perceived burden and associated factors in Chinese adult epilepsy patients: a cross-sectional study. Front Neurol. (2022) 13:994664. doi: 10.3389/fneur.2022.994664, PMID: 36176558 PMC9513448

[46] Zhong R Zhang H Chen Q Guo X Han Y Lin W. Social isolation and associated factors in Chinese adults with epilepsy: a cross-sectional study. Front Neurol. (2021) 12:813698. doi: 10.3389/fneur.2021.813698, PMID: 35087477 PMC8787157

[47] Castro D Ferreira F de Castro I Rodrigues AR Correia M Ribeiro J. The differential role of central and bridge symptoms in deactivating psychopathological networks. Front Psychol. (2019) 10:2448. doi: 10.3389/fpsyg.2019.02448, PMID: 31827450 PMC6849493

[48] Steinbrenner M Tito T Dehnicke C Holtkamp M. Predictors and reasons for epilepsy patients to decline surgery: a prospective study. J Neurol. (2023) 270:2302–7. doi: 10.1007/s00415-022-11510-3, PMID: 36473975 PMC10025225

[49] Chesi P Marini MG Scarlata P Mecarelli O Aguglia U Assenza G. Epileptologists telling their experiences caring for patients with epilepsy. Seizure. (2021) 85:19–25. doi: 10.1016/j.seizure.2020.12.012, PMID: 33385785

[50] Ertan D Hubert-Jacquot C Maillard L Sanchez S Jansen C Fracomme L. Anticipatory anxiety of epileptic seizures: an overlooked dimension linked to trauma history. Seizure. (2021) 85:64–9. doi: 10.1016/j.seizure.2020.12.006, PMID: 33444881

[51] Lai ST Lim KS Tang V Low WY. Mindfulness-based intervention to promote psychological wellbeing in people with epilepsy: a randomized controlled trial. Epilepsy Behav. (2021) 118:107916. doi: 10.1016/j.yebeh.2021.107916, PMID: 33743343

[52] Alemu A Dendir G Gonfa A Sisay Y Tadesse T Abebe A. Health-related quality of life and associated factors among adult patients with epilepsy in public hospitals of Wolaita zone, southern Ethiopia. An embedded mixed method study. Epilepsy Behav. (2023) 145:109316. doi: 10.1016/j.yebeh.2023.109316, PMID: 37356224

[53] Addis B Wolde M Minyihun A Aschalew AY. Prevalence of depression and associated factors among patients with epilepsy at the University of Gondar Comprehensive Specialized Hospital, Northwest Ethiopia, 2019. PLoS One. (2021) 16:e0257942. doi: 10.1371/journal.pone.0257942, PMID: 34695130 PMC8544874

