# Supplementary material for: Fear of disease in patients with epilepsy – a network analysis
Source: Front Neurol. 2024 Mar 7;15:1285744. doi: 10.3389/fneur.2024.1285744 (PMC10954812; doi:10.3389/fneur.2024.1285744)
Supplement: Supplementary file 1 [file Data_Sheet_1.docx]

**
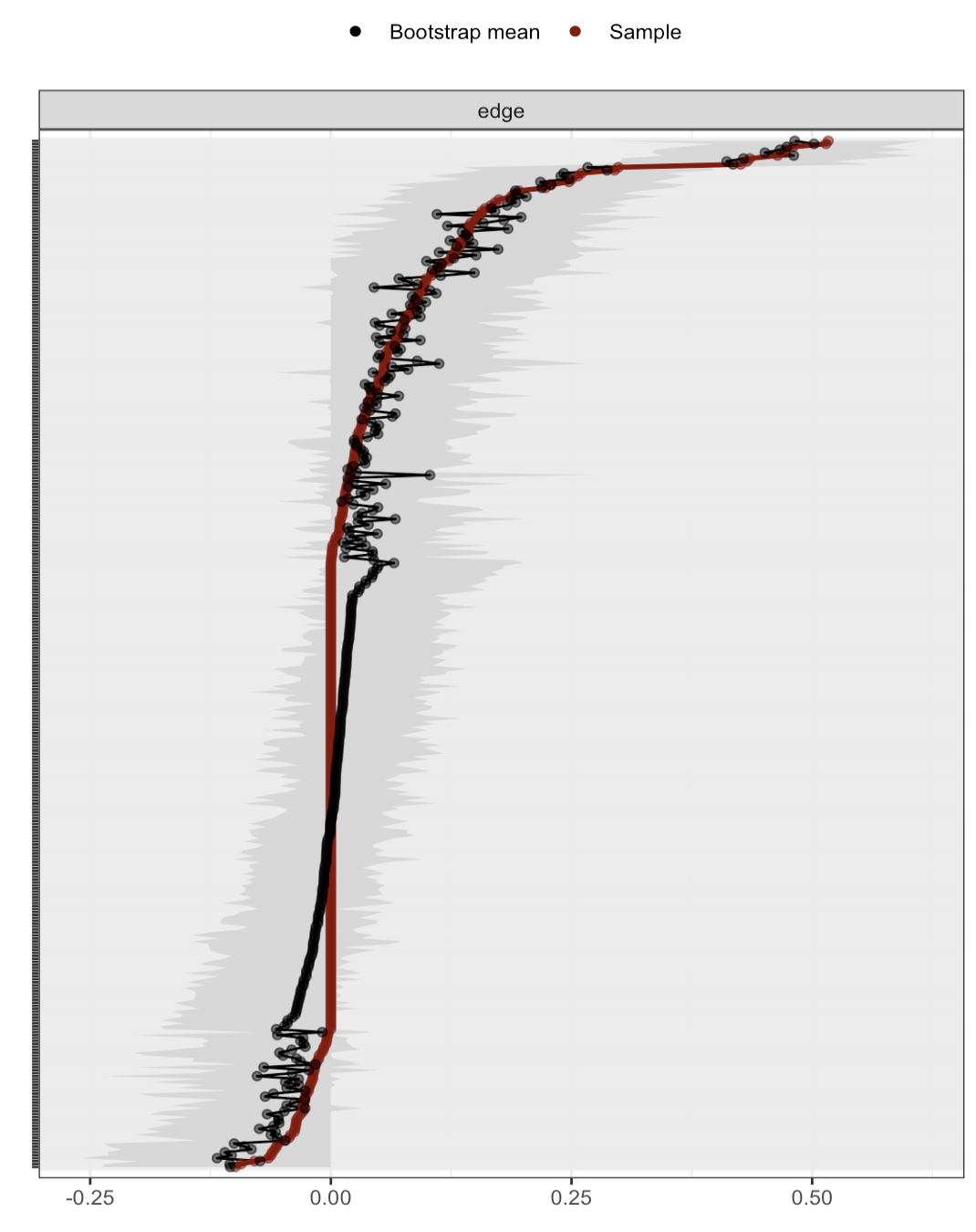
**

**Figure S1. Bootstrapped 95%CIs of estimated edge weights**

*Note*: The red line depicts the sample edge weights and the gray bar depicts the bootstrapped confidence interval.


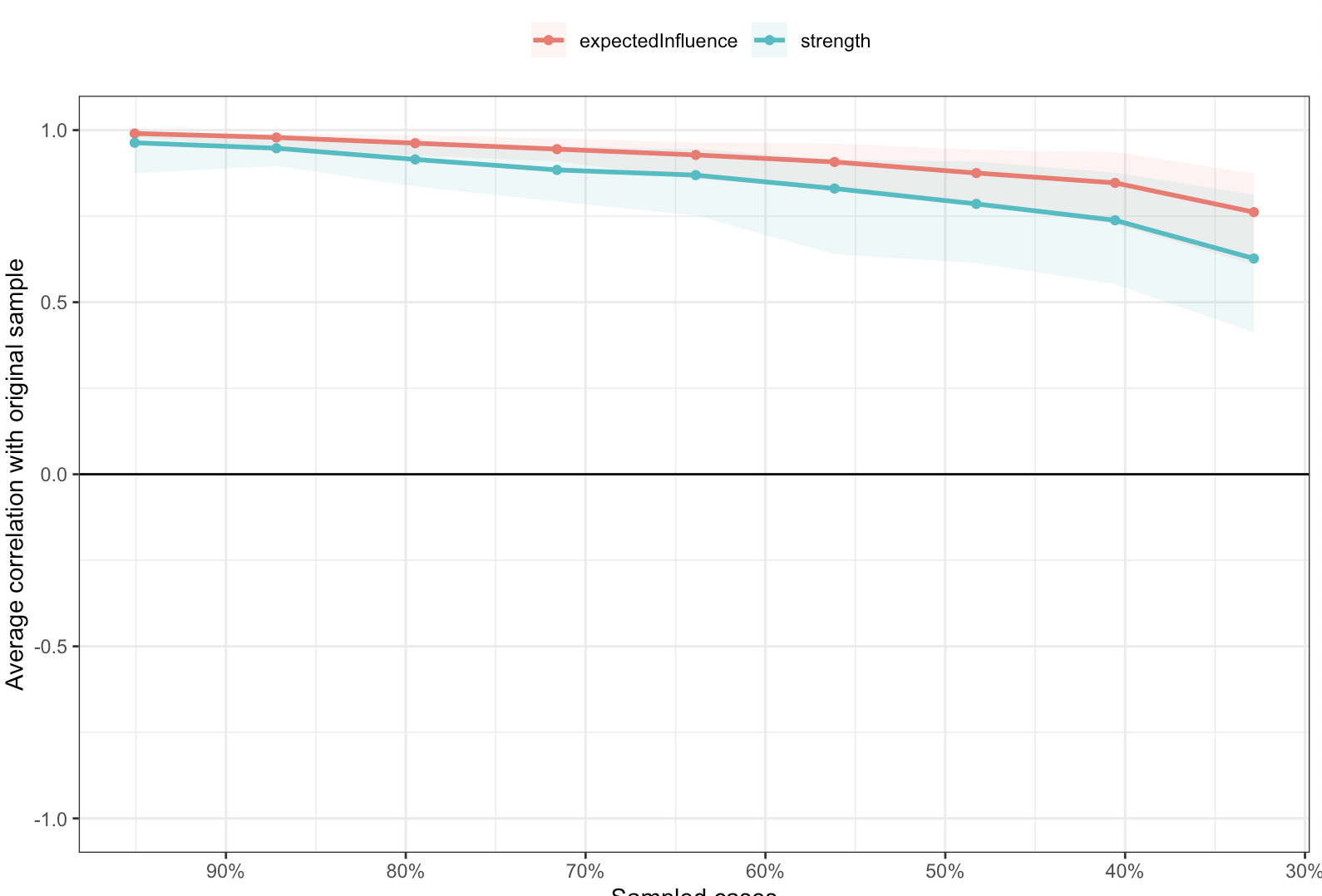


**Figure S2. Stability of centrality indices by case dropping subset**

**
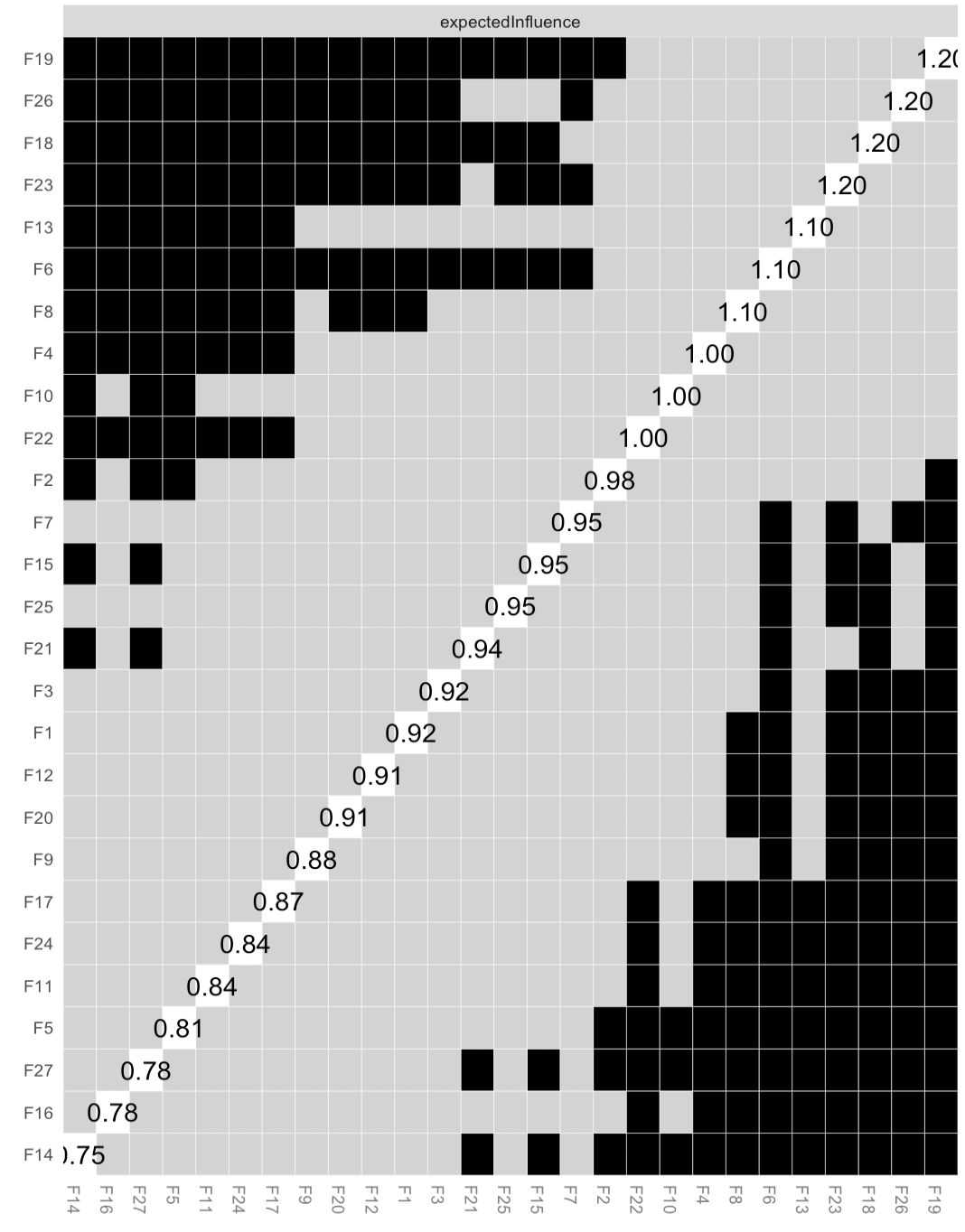
**

**Figure S3. Bootstrapped difference test for expected influences**

*Note:* Gray boxes indicate expected influences that do not differ significantly from one another, while black boxes indicate node expected influences that do differ significantly. The number in the white boxes (i.e., diagonal line) represent the value of node expected influences.

**
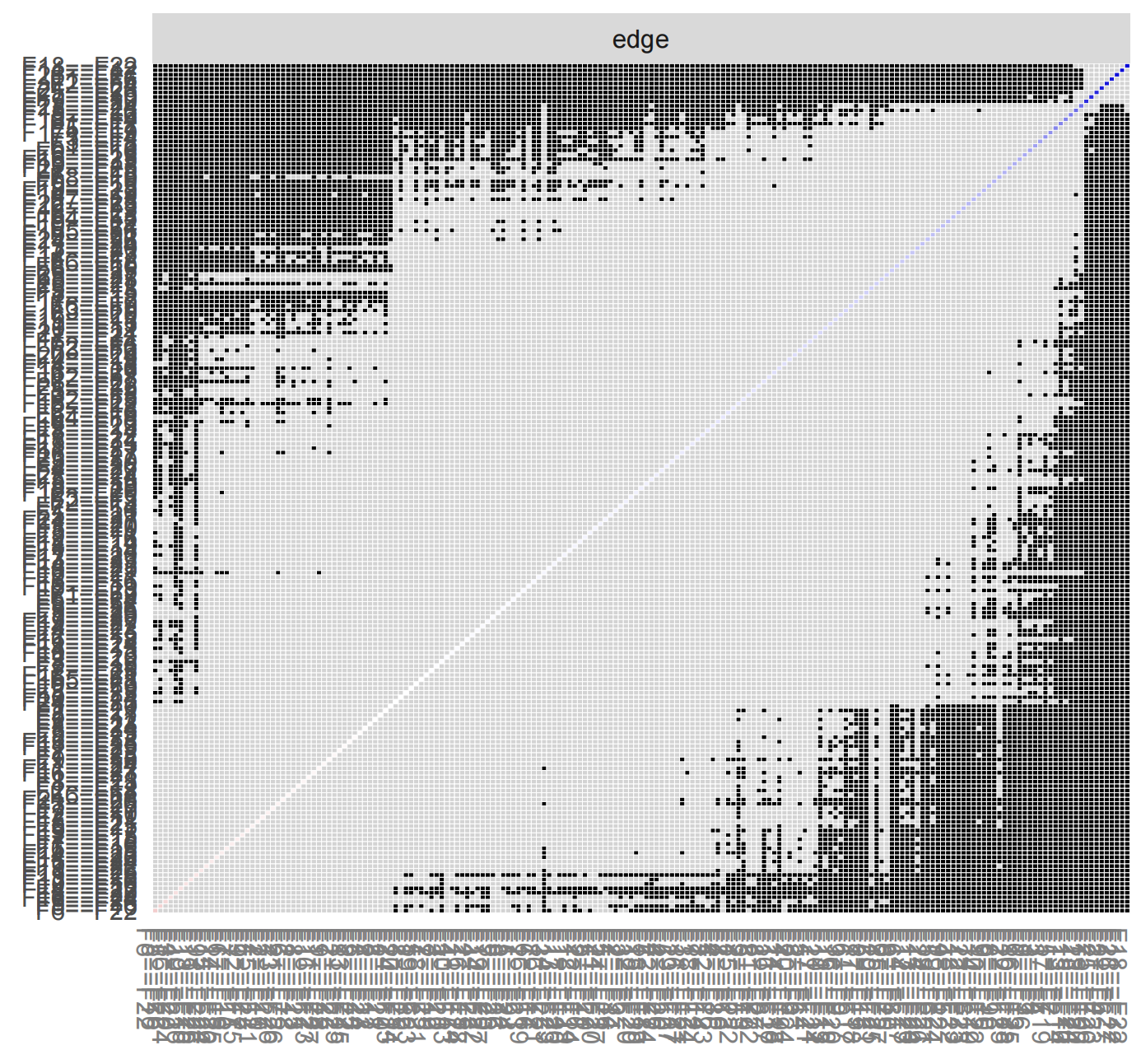
**

**Figure S4. Bootstrapped difference test for edge weights**

*Note:* Gray boxes indicate edge weights that do not differ significantly from one another, while black boxes indicate edge weights that do differ significantly. Blue and red boxes on the diagonal correspond to edge weights with positive and negative correlations, respectively.

**Table S1 The validity results of the Edge Invariance Test**

| Item | Item | p-value | Test statistic |
| --- | --- | --- | --- |
| C1-F2 | C1-F3 | 0.00990099 | 0.19338195 |
| C1-F8 | C4-F10 | 0.00990099 | 0.20619805 |
| C2-F13 | C5-F19 | 0.00990099 | 0.20320032 |
| C4-F10 | C5-F21 | 0.00990099 | 0.04075083 |
| C2-F13 | C4-F22 | 0.00990099 | 0.17011199 |
| C3-F15 | C4-F22 | 0.00990099 | 0.11218326 |
| C5-F19 | C4-F22 | 0.00990099 | 0.04615183 |
| C5-F19 | C5-F24 | 0.00990099 | 0.20457057 |
| C1-F6 | C5-F20 | 0.01980198 | 0.05682792 |
| C2-F12 | C5-F20 | 0.01980198 | 0.10992361 |
| C3-F16 | C5-F20 | 0.01980198 | 0.10038145 |
| C1-F9 | C3-F26 | 0.01980198 | 0.04467838 |
| C2-F11 | C3-F27 | 0.01980198 | 0.04655578 |
| C1-F4 | C5-F17 | 0.02970297 | 0.00333165 |
| C3-F16 | C5-F17 | 0.02970297 | 0.05552683 |
| C1-F2 | C5-F21 | 0.02970297 | 0.01554736 |
| C5-F21 | C3-F26 | 0.02970297 | 0.01442378 |
| C1-F3 | C3-F27 | 0.02970297 | 0.08553444 |
| C1-F4 | C5-F24 | 0.03960396 | 0.02160587 |
| C4-F7 | C5-F24 | 0.03960396 | 0.00368913 |
| C4-F7 | C3-F25 | 0.03960396 | 0.03207183 |
| C1-F4 | C4-F10 | 0.04950495 | 0.01954959 |
| C2-F11 | C3-F16 | 0.04950495 | 0.03494345 |
| C3-F15 | C3-F16 | 0.04950495 | 0.13048667 |
| C4-F10 | C5-F23 | 0.04950495 | 0.07828991 |
